# What’s Next for the Standard Short-Course Regimen for Treatment of Multidrug-Resistant Tuberculosis

**DOI:** 10.4269/ajtmh.18-0985

**Published:** 2019-01-14

**Authors:** Jonathon R. Campbell, Dick Menzies

**Affiliations:** McGill International TB Centre, Montreal, Canada

In August 2018, the WHO published preliminary recommendations that called for a radical change in the treatment of multidrug-resistant tuberculosis (MDR-TB).^1^ The newly proposed first-line regimen would be all-oral, consisting of linezolid, bedaquiline, moxifloxacin/levofloxacin, plus clofazimine, and/or cycloserine/terizidone. The second-line injectable agents of kanamycin and capreomycin are no longer recommended because of evidence of limited benefit, and amikacin is not recommended as part of the first-line regimen for treatment of MDR because of its poor safety and tolerability.^1,2^ In addition to superior effectiveness, and likely better tolerability, the new all-oral regimen has the important additional advantage of containing two or three drugs to which isolates should be fully susceptible, reducing the risk of starting ineffective treatment while awaiting results of susceptibility testing to second-line drugs (SLD). The many advantages of this new regimen cast uncertainty on the role of the standardized short-course MDR treatment (STR). This regimen, consisting of 4–6 months of kanamycin, high-dose isoniazid, ethambutol, pyrazinamide, moxifloxacin/gatifloxacin, clofazimine, and prothionamide, followed by 5 months of ethambutol, pyrazinamide, moxifloxacin/gatifloxacin, and clofazimine, has lower rates of loss to follow-up than are reported for the longer injection-based regimens.^3^

In this issue of the *AJTMH*, Walsh et al. report high rates of resistance to several drugs used in the STR based on drug susceptibility testing (DST) of isolates from 239 consecutive MDR-TB patients diagnosed between 2008 and 2015 at the GHESKIO clinic in Haiti.^4^ They found resistance in 95% of patients to high-dose isoniazid, 57% to pyrazinamide, 77% to ethambutol, and 16% to ethionamide. Very few isolates were resistant to fluoroquinolones or second-line injectables (3%), and few patients had previous exposure to SLD (0.4%), which are considered contraindications for use of the STR in current WHO recommendations. Hence, only a small fraction of individuals would have been excluded from the STR and so many would have received the STR despite disease with isolates resistant to pyrazinamide and ethambutol. Furthermore, based on drug susceptibility patterns, the authors predicted that only 118 (49.2%) would have received at least four effective drugs in the initial phase of therapy and at least three effective drugs in the continuation phase. The authors conclude that empiric use of the STR in their setting would entail an unacceptably high risk of failure due to high prevalence of resistance to components of the STR.

Although excellent results can be achieved with short regimens in patients who are susceptible to all drugs in the regimen,^3,5,6^ these studies and recent evidence demonstrate trends for increased risk of treatment failure if resistance is present for drugs that would not exclude a patient from receiving the STR, including pyrazinamide, ethambutol, and ethionamide.^1,7,8^ Despite concerns about the reliability of DST for ethambutol, pyrazinamide, and many of the SLD, use of these drugs despite in vitro resistance was associated with consistently worse outcomes, compared with those with sensitive organisms, in a recently published individual patient data meta-analysis of 13,000 patients who received “traditional” duration MDR-TB treatment.^9^ In summary, all evidence suggests that the use of drugs despite in vitro resistance results in poor outcomes for patients; hence, continuing a standardized regimen despite resistance to any of the component drugs (assuming availability of other effective drugs) would be contrary to fundamental principles of TB care. This means that empiric treatment with standardized regimens will be acceptable only in settings with very low prevalence of resistance to the component drugs of the regimen. In settings such as Haiti, the STR is unlikely to provide acceptable results unless DST can be performed quickly, and the regimen modified promptly thereafter. For most SLD, as well as ethambutol and pyrazinamide, there are no WHO endorsed rapid molecular tests to detect resistance.^10,11^ Hence, patients receiving the STR may receive an inadequate regimen for 2–3 months until results of DST are available, increasing the risk of worsening morbidity and amplification of resistance. Evidence is needed on the outcomes of patients who initiate STR and require modification of regimen due to resistance to component drugs detected by DST. In areas where empiric treatment with the STR persists, it is imperative for programs to carefully document outcomes in patients switched from the STR in the face of drug resistance to know the true consequences of this practice.

There is an urgent need for shorter MDR-TB regimens. Although the new all-oral regimen recommended by the WHO is very promising in terms of efficacy and tolerability, the optimal duration is unclear. This can be answered best with large-scale randomized trials. Some are ongoing, but these will take many years.^12,13^ In the meantime, we suggest that well-characterized cohorts of patients could receive progressively shorter durations of this regimen—with careful monitoring of tolerability, safety, adherence, and efficacy during treatment. This method was used very successfully in Bangladesh to define the optimal duration and composition of the current STR.^3^ In our opinion, the most important outcome to define optimal duration will be microbiologically confirmed relapse in the first year after the end of therapy. The current STR may provide good results in specific populations where the prevalence of resistance to ethambutol, pyrazinamide, and SLD is low. In most of the settings, however, this regimen will be of limited utility, unless DST to these drugs can be performed, and regimens adjusted rapidly—and without harm to patients.
